# Emerging Roles of Lysophosphatidic Acid in Macrophages and Inflammatory Diseases

**DOI:** 10.3390/ijms241512524

**Published:** 2023-08-07

**Authors:** Shufan Jiang, Huili Yang, Mingqing Li

**Affiliations:** 1 Laboratory for Reproductive Immunology, Hospital of Obstetrics and Gynecology, Fudan University, Shanghai 200080, China; 21301050053@m.fudan.edu.cn; 2 Shanghai Medical College, Fudan University, Shanghai 200032, China; 3 Shanghai Key Laboratory of Female Reproductive Endocrine Related Diseases, Hospital of Obstetrics and Gynecology, Fudan University, Shanghai 200080, China

**Keywords:** lysophosphatidic acid, lysophosphatidic acid receptor, macrophage, inflammation, atherosclerosis, fibrosis

## Abstract

Lysophosphatidic acid (LPA) is a bioactive phospholipid that regulates physiological and pathological processes in numerous cell biological functions, including cell migration, apoptosis, and proliferation. Macrophages are found in most human tissues and have multiple physiological and pathological functions. There is growing evidence that LPA signaling plays a significant role in the physiological function of macrophages and accelerates the development of diseases caused by macrophage dysfunction and inflammation, such as inflammation-related diseases, cancer, atherosclerosis, and fibrosis. In this review, we summarize the roles of LPA in macrophages, analyze numerous macrophage- and inflammation-associated diseases triggered by LPA, and discuss LPA-targeting therapeutic strategies.

## 1. Introduction

Macrophages are a significant component of the innate immune system and are found in nearly all human tissues. It is now known that macrophages serve multiple purposes in both physiological and pathophysiological contexts. These functions include development, homeostasis, repair, and pathogen-specific immune responses [1]. However, continuous insult can disrupt homeostasis and repair, leading to macrophage-related diseases such as inflammation, atherosclerosis, and fibrosis.

Lysophosphatidic acid (LPA) is a bioactive lipid identified in various tissues and cells and works via six different types of G-protein-coupled receptors (GPCRs). LPA acts via specific receptors (LPA_1_–LPA_6_) and is related to a wide range of cell responses, such as proliferation and migration [2]. In recent years, the physiological and pathological relationship between LPA and macrophages has become increasingly evident. In our review, we cover LPA metabolism. In addition, we focus on the physiological functions of macrophages mediated by LPA and the numerous macrophage-associated LPA-related diseases. Finally, we summarize the therapeutic potential of pharmacologically targeting LPA.

## 2. The Metabolism of LPA

**Figure 1:**
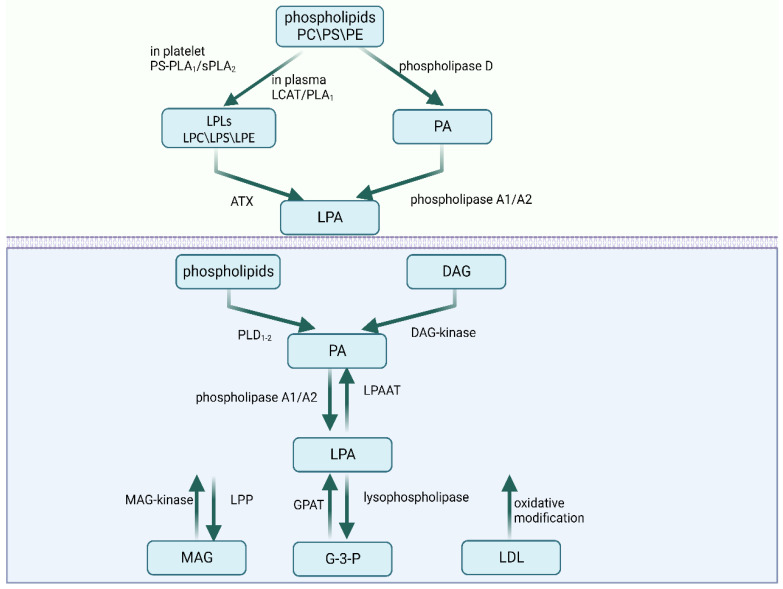
The extracellular and intracellular metabolism of LPA. Abbreviations: PC—phosphatidylcholine; PS—phosphatidylserine; PE—phosphatidylethanolamine; PS-PLA_1_—phosphatidylserine-specific phospholipase A_1_; sPLA_2_—secretory phospholipase A_2_; LCAT—lecithin–cholesterolacyltransferase; LPLs—lysophospholipids; LPC—lysophosphatidylcholine; LPS—lysophosphatidylserine; LPE—lysophosphatidylethanolamine; ATX—autotaxin; DAG—diacylglycerol; PLD—phospholipase D; LPAAT—LPA-acyltransferase; MAG—monoacylglycerol; GPAT—glycerophosphate acyltransferase; G-3-P—glycerol 3-phosphate; LDL—low-density lipoprotein. Created with BioRender.com (accessed on 28 July 2023).

LPA molecules‘ characteristics comprise a glycerol backbone with a phosphate group in the sn-3 position, a fatty acid chain, and a hydroxyl group in the sn-1 or sn-2 position. Saturated (16:0 and 18:0) and unsaturated (16:1, 18:1, 18:2, and 20:4) fatty acids make up the LPA species. Different LPAs have distinctive biological behaviors. For example, LPA 20:4 promotes the development of macrophages and the spread of plaques, but LPA 18:0 does not. According to research conducted by Zhou et al., the mitogenic effects of LPA 18:0, 18:2, and 18:3 are also different [3]. In addition, LPA is produced via the internal and extracellular production of cell membranes (Figure 1).

### 2.1. Extracellular Synthesis Pathways

LPA can be extracellularly synthesized mainly via two pathways. In the first pathway, the membrane phospholipids phosphatidylcholine, phosphatidylserine, and phosphatidylethanolamine are converted to corresponding lysophospholipids (LPLs) including lysophosphatidylcholine (LPC), lysophosphatidylserine (LPS), and lysophosphatidylethanolamine (LPE). However, the enzymes catalyzing the process vary depending on the environment. In plasma, LPC is produced via lecithin–cholesterolacyltransferase (LCAT) and phospholipase A_1_ activity. In rats’ platelets, the conversion is accomplished by phosphatidylserine-specific phospholipase A_1_ (PS-PLA_1_) or secretory phospholipase A_2_ (sPLA_2_), while in humans, the conversion happens on the plasma membrane [4,5]. In both contexts, autotaxin (ATX) then converts LPLs to LPA. LPLs can elicit a variety of cell responses by activating GPCRs that are particular to each type of LPL. ATX, also known as ectonucleotide pyrophosphatase/phosphodiesterase 2 (ENPP2), is a glycoprotein with lysophospholipase D activity that is secreted from cells [6]. ATX is also a significant contributor to extracellular LPA. Plasma LPA levels decreased by approximately half in ATX heterozygous mice [7]. LPL mediators include lysophosphatidylserine (LysoPS), sphingosine 1-phosphate (S1P), and LPA. In the second pathway, phospholipase D initially converts phospholipids to PA. Then, PA is directly transformed into LPA via the activities of phospholipase A_1_ or phospholipase A_2_ [2].

### 2.2. Intracellular Synthesis Pathways

A minimum of four intracellular synthesis routes have been identified. Initially, the monoacylglycerol kinase (MAGK) pathway uses monoacylglycerol kinase (MAG-kinase) to phosphorylate monoacylglycerol (MAG) into LPA [8]. LPA can also be synthesized in the endoplasmic reticulum and mitochondria. The glycerophosphate acyltransferase (GPAT) in these organelles can form LPA via the acylation of glycerol 3-phosphate (G-3-P). The third pathway is initiated via the production of PA from phospholipids by phospholipase D (PLD_1–2_) or from diacylglycerol (DAG) by diacylglycerol kinase (DAG-kinase). Then, PA is converted into LPA by phospholipase A_1_ or phospholipase A_2._ The difference between phospholipase A_1_ and phospholipase A_2_ is that phospholipase A_1_ produces 2-acyl-LPA, while phospholipase A_2_ produces 1-acyl-LPA [8]. Finally, low-density lipoprotein (LDL) can also produce LPA via oxidative modification.

### 2.3. Degradation

Several enzymes, including LPA-acyltransferase (LPAAT), lipid phosphate phosphatases (LPPs), and lysophospholipase, are capable of degrading LPA. LPA may be converted to PA by LPAAT, generating MAG via LPP [9], or converted to G-3-P by lysophospholipase [10].

## 3. LPA Signaling and Receptors

Six currently recognized LPA receptors, LPA_1–6_, mediate the numerous physiological effects of LPA. The protein names are LPA_1–6_, and the gene names are *LPAR1–6* (human) and *Lpar1–6* (non-human). These GPCRs couple to G_12/13_, G_q/11_, G_i/o_, and G_s_ and initiate various signaling cascades (Table 1).

LPA_1_ was the first-identified LPA receptor. The *LPAR1* gene is widely expressed in various organs, including the testis, lungs, brain, heart, spleen, small intestine, thymus, stomach, and skeletal muscle [17]. LPA_1_ can also serve as a marker of stem and progenitor cells in the dentate gyrus, which outperforms the current gold standard, nestin [29]. Immune organs express LPA_1_ and LPA_2,_ and depending on their activity levels, LPA may either increase or decrease the activity of T cells. LPA_2_ prevents T cells from secreting interleukin (IL)-2 when they are not activated. LPA_1_ is elevated in activated T cells, whereas LPA_2_ is downregulated, and when IL-2 production is triggered, LPA_1_ and LPA_2_’s antagonistic effects on T cells are visible [2]. LPA_1_ and LPA_2_ are also able to work corporately. LPA_1_ and LPA_2_ induce the phosphorylation of the ezrin/radixin/moesin (ERM) proteins at their C-termini. The LPA_1_/LPA_2_/ERM pathway can stimulate the migration of ovarian cancer cells [30]. *LPAR3* is strongly expressed in the human testis, heart, prostate, and pancreas and less expressed in human lungs and ovaries [31]. LPA_1–3_ belong to the EDG family of LPA receptors.

After the discovery of the EDG family of LPA receptors, the non-EDG family of LPA receptors, LPA_4–6_, was discovered and provided an additional framework for comprehending LPA signaling. LPA_4_ is the first LPA receptor that shows a dissimilar sequence compared with the formerly discovered receptors, LPA_1–3_. LPA_4_ is more related to P2Y purinergic receptors but does not respond to any nucleotides or nucleosides [23]. In humans, *LPAR4* is prominently expressed in the ovaries and less prominently in the colon, spleen, testis, prostate, small intestine, heart, brain, thymus, and pancreas [2]. LPA_5_ is an orphan GPCR (GPR92). *LPAR5* is highly expressed in the spleen and less expressed in the small intestine, heart, placenta, colon, and liver [8]. In addition, diffused LPA_5_ expression has also been observed in the developing brain, suggesting LPA_5_ may participate in brain development [32]. LPA_6_ is the newest identified LPA receptor. The understanding of LPA_6_ remains limited. LPA_6_ is implicated in the metastasis of androgen-independent prostate cancer cells [28] and hypotrichosis simplex. The recent determination of the crystal structure of LPA_6_ explains the ligand recognition mechanism of the non-EDG family of LPA receptors [33].

Besides LPA_1–6_, the GPR87 and P2Y10 receptors are also known as LPA receptors. Furthermore, LPA stimuli activate the transient receptor potential vanilloid 1 (TRPV1) ion channel [8]. The peroxisome proliferator-activated receptor γ (PPARγ) is another intracellular receptor for LPA. PPARγ is expressed in monocytes and macrophages and regulates various physiological or pathological activities, such as atherosclerosis, inflammation, and fibrosis [34,35].

## 4. LPA, Macrophages, and Inflammation

### 4.1. LPA in the Migration and Infiltration of Macrophages

LPA_1–4_ are expressed in macrophages. LPA_1_ is highly expressed in monocytes but decreases during differentiation [36]. LPA_5_ expression has been discovered in macrophages. Various LPA signaling pathways have been revealed to be associated with the migration and penetration of macrophages. A previous study showed that LPA is a significant survival factor for macrophages. LPA is a key noncytokine survival factor in serum, and it functions via phosphatidylinositol 3-kinase (PI3K) to inhibit apoptosis [37]. Tyrosine kinase can control the RhoA signaling pathway when LPA is present. Worthylake et al. observed that inhibiting RhoA in monocytes limits their transendothelial migration and, consequently, their transition into tissue macrophages. The activation of RhoA and p160ROCK, a downstream effector of RhoA, is necessary for monocyte rearward migration [38]. In addition, a recent study showed that LPA increased a chronic inflammatory milieu in the rotator cuff (RC) muscle, improved RhoA signaling, caused macrophage infiltration, and subsequently accelerated RC muscle fibrosis, fatty infiltration, and atrophy [39].

Additionally, LPA is involved in atherosclerosis (AS). In the subendothelial area, monocyte-derived mononuclear phagocytes consume normal and modified subendothelial lipoprotein and subsequently transform into cholesterol-laden foam cells that remain in plaques and accelerate the course of the illness [40]. The advancement of AS is related to the increase in monocyte-derived cells in AS plaque, which is caused by the recruitment of monocytes into subendothelial areas and the reduced rate of their migratory clearance from lesions [41]. A preliminary study revealed that LPA inhibits the combination of HNF1 and the Fut8 promoter region by activating the LPA_1_ and LPA_3_ receptors of foam cells, and LPA, therefore, reduces the migratory capacity of foam cells [42].

Microglia is one kind of tissue macrophage. In the central nervous system (CNS), microglia can express high ATX levels and are considered the primary source of LPA in the CNS [43]. The expression of LPA_1_ and ATX in glioblastomas (GBMs) correlates with glioma aggressiveness and predicts a poor prognosis. LPA derived from microglia promotes GBM cell migration, survival, and proliferation via LPA_1_. GBM can induce microglia to produce more LPA, and this positive feedback may accelerate tumor growth [44]. Research has shown that a hypoxic environment may also enhance the effects of the ATX-LPA-LPA_1_ axis [44,45]; therefore, a hypoxic microenvironment is one of the characteristics of rapidly growing malignancies such as GBMs [46].

### 4.2. LPA in Inflammation Regulation of Macrophages

Inflammation is a defensive physiological reaction to pathogens, particles, and damaged tissues. Inflammation usually has a beneficial effect but also causes collateral harm to neighboring cells. Therefore, inflammation is a significant factor in increasing an existing illness state [47]. The ATX/LPA signaling pathway is involved in the response to inflammation. During typical tissue remodeling and wound healing, ATX/LPA signals may cause platelet aggregation and promote the growth and migration of keratinocytes, vascular smooth muscle cells, fibroblasts, and endothelial cells [48]. Notably, ATX/LPA signals exacerbate chronic inflammation in chronic pathological situations by generating cytokines and attracting inflammatory cells into the local tissue environment [49]. Tang et al. observed that doxycycline inhibits NF-κB activation and reduces plasma LPA concentrations to reduce inflammation caused by breast cancer. According to the literature, LPA can facilitate the nuclear translocation of NF-κB, hence boosting the production of inflammatory cytokines. Moreover, high LPA concentrations exacerbate tumor inflammation [50].

LPA directly promotes the release of proinflammatory mediators, such as IL-1 and reactive oxygen species (ROS), in macrophages [51]. In vitro studies also exhibited that LPA could activate LPS-induced nucleotide-binding oligomerization domain-like receptor family pyrin-domain-containing 3 (NLRP3) inflammasome via LPA_5_, and LPA_5_ signaling was found to upregulate the NLRP3 expression in psoriasis lesions [52]. The NLRP3 inflammasome induces the production of the proinflammatory cytokines IL-1 and IL-18. Recent research has shown that LPA_1_ can activate the NLRP3 inflammasome via ERK_1/2_ and p38, and the activated NLRP3 inflammasome is implicated in ischemic brain damage [53].

Nonetheless, several studies have indicated that LPA may inhibit inflammation. Exogenous LPA demonstrated a protective effect against bacterial-endotoxemia-induced kidney inflammation and impairment [54]. LPS can activate inflammation cascades in macrophages. Moreover, LPS mediates the cyclooxygenase-2 (COX-2)/prostaglandin E2 (PGE_2_) pathway and the inducible NO synthase (iNOS)/NO pathway. LPA has an anti-inflammatory role via G_αi_ owing to its suppressive effect on LPS-induced inflammation due to p38, NF-κB, and Akt [55]. Additionally, in primary macrophages and macrophage-like J774 cells, LPS-induced inflammation can be deregulated via LPA signaling mediated by LPA_5_ and LPA_6_ [56]. Different LPS doses, macrophage treatments, and cell types may be responsible for discrepancies.

## 5. ATX/LPA Signals in Macrophage Dysfunction and Inflammation Diseases

### 5.1. Autoimmune Encephalomyelitis

**Figure 2:**
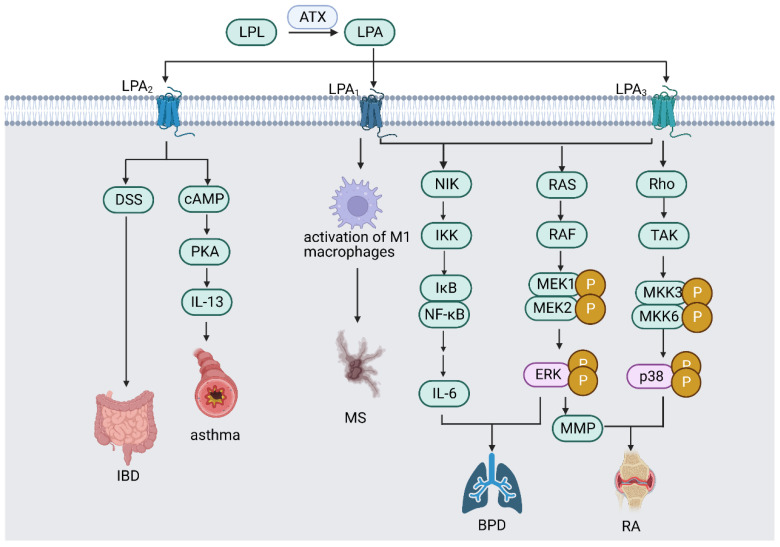
ATX/LPA signaling pathway in different inflammatory diseases. ATX/LPA signaling pathway mediates various inflammatory diseases through LPA receptors. LPA_1_ promotes MS by activating M1 macrophages; LPA_2_ worsens DSS-induced IBD; LPA_2_ causes asthma by enhancing IL-13 expression via activating PKA signaling pathway; LPA_1_ and LPA_3_ promote BPD by producing IL-6 via activating NF-κB signaling pathway; LPA_1_ and LPA_3_ activate ERK signaling pathway, which promotes BPD and worsens RA via producing MMP; and LPA_3_ mediates the pathogenesis of RA by activating p38 MAPK signaling pathway. (Abbreviations: LPL—lysophospholipid; ATX—autotaxin; LPA—lysophosphatidic acid; MS—multiple sclerosis; DSS—dextran sulfate sodium; IBD—inflammatory bowel disease; cAMP—cyclic adenosine 3,5-monophosphate; PKA—protein kinase A; IL—interleukin; NIK—NF-κB-inducing kinase; IKK—IκB kinase; NF-κB—nuclear factor κB; ERK—extracellular-signal-regulated kinase; MMP—matrix metalloproteinase; TAK—transforming growth-factor-β-activated kinase; MKK—mitogen-activated protein kinase kinase; BPD—bronchopulmonary dysplasia; RA—rheumatoid arthritis). Created with Biorender.com (accessed on 28 July 2023).

As the most common form of persistent inflammation of the central nervous system (CNS), multiple sclerosis (MS) is characterized by inflammation, demyelination, and glial response in the brain and spinal cord, reversible neurologic impairments, and decreased cognition and movement [57]. MS pathogenesis can be derived from the experimental autoimmune encephalomyelitis model, which can be generated in immunological animals by exposing them to myelin antigens [58]. ATX-expressing F4/80^+^ CD11b^+^ cells, primarily activated microglia and macrophages, are a hallmark of autoimmune encephalomyelitis. In addition, ATX genetic deletion from CD11b^+^ cells inhibits the progression of autoimmune encephalomyelitis, indicating the potential therapeutic value of ATX targeting [59]. Moreover, LPA_1_ expression is associated with a pro-inflammatory phenotype of macrophages and contributes to the development of MS and experimental autoimmune encephalomyelitis (EAE) (Figure 2), indicating LPA_1_ as a therapeutic target biomarker for MS and EAE [60].

### 5.2. Infection of the Gastrointestinal Tract

Macrophages are crucial for maintaining intestinal homeostasis and intestinal immunity. Nonetheless, they can also cause chronic illnesses of the gastrointestinal system, such as inflammatory bowel disease (IBD) [61]. ATX mRNA expression was elevated in the inflamed mucosa of IBD patients compared with healthy individuals [62]. A recent study has demonstrated that ATX has potent proinflammatory effects in colitis. The ATX/LPA axis worsens dextran sulfate sodium (DSS)-induced colitis by activating the LPA_2_ receptor in macrophages and is a possible therapeutic target for IBD [63]. However, a study on myeloid-cell-lineage-restricted ATX knockout mice showed that ATX deficiency impairs Toll-like receptor 4 (TLR4)-mediated responses in macrophages and hampers the innate immune response, leading to the accelerated development of colitis [64]. The study indicates that the inhibition of ATX may also impair the immune response, which needs further research to overcome the therapeutical barrier.

### 5.3. Asthma

Asthma is often characterized by chronic inflammation of the airways. Inflammation is linked to hyper-responsiveness, which causes shortness of breath, chest constriction, and dyspnea [65]. Traditional treatments for asthma include utilizing corticosteroids, but novel methods are required to overcome steroid-induced side effects [66]. LPA is present in human BAL fluid at baseline and is more prevalent after allergic inflammation [67]. Additionally, increased ATX is present in asthmatic BAL fluid. Elevated ATX is associated with increased 22:5 and 22:6 LPA levels [68]. LPA can also selectively enhance IL-13 expression by activating the cAMP signaling pathway [69]. Moreover, LPA induces actin reorganization, chemotaxis, and calcium mobilization in human eosinophils [70]. Therefore, LPA may promote the release of Th2 cell cytokines in asthmatic airway inflammation.

Asthma therapy has been found to target LPA_2_. When administered before an antigen challenge or prior to sensitization, the LPA_2_ antagonist H2L5186303 efficiently inhibits symptoms and immunological responses in BALA/c mice [71]. LPA upregulates the release of proinflammatory cytokines (such as IL-8) and PGE2 and attenuates the effect of Th2-type cytokines (such as IL-13). In contrast, some studies have shown that LPA could be an anti-inflammation mediator. Mice lacking the LPA_2_ gene (*Lpar*_2_^−/−^) had greater lung inflammation than wild-type mice [72]. Such discrepancies may result from the differences between mouse models of asthma or the recruited cell types [73].

### 5.4. Rheumatoid Arthritis (RA)

RA, as a prevalent autoimmune disorder, is characterized by synovial inflammation and autoantibody production, hyperplasia, and cartilage and bone degradation [74]. Resident fibroblast-like synovial cells (FLS) and osteoblasts may promote the disease [75,76]. FLS is a major source of metalloproteinases and proinflammatory mediators and causes RA joint functional disability. The activation of osteoblasts and the presence of osteoclasts also result in permanent joint deformities and impairments in RA [77]. LPA signaling is deeply involved in the development of RA. LPA plays a prominent part in promoting the production of cyclooxygenase-2 (COX-2) with inflammatory cytokines [78]. Moreover, LPA induces the migration of FLS and the secretion of IL-8 and IL-6. LPA_1_ and LPA_3_ also mediate various pathways contributing to the pathogenesis of RA [79]. For instance, LPA_1_ transcriptionally increases matrix metalloproteinase (MMP) production by stimulating the LPA_1_/ERK_1/2_ signaling pathway. Additionally, tumor necrosis factor (TNF) increases LPA_3_ expression in RA patients, which modulates cytokine production via p38 MAPK and Rho kinase [79]. TNF can also drive ATX expression in the synovium. Anti-TNF treatment with infliximab injection has been shown to attenuate ATX expression [80].

LPA receptors may be attractive therapeutic targets for the treatment of RA pathophysiology. Orosa et al. found that Ki16425, a selective antagonist for LPA_1/3_ receptors, was an effective therapy for the K/BxN serum transfer model of arthritis [81]. Recent research has revealed that berberine can inhibit the inflammatory proliferation of FLS by modulating severe signaling pathways, including LPA/LPA_1_/ERK/p38 MAPK and thus prevent cartilage and bone destruction [82]. Therefore, berberine can serve as a novel therapeutical drug for RA treatment.

### 5.5. Neonatal Chronic Lung Disease or Bronchopulmonary Dysplasia (BPD)

BPD is the most common chronic respiratory disease in infants. Mechanical ventilation, oxidant injury, and proinflammation mediators may cause barotrauma or volutrauma [83]. Studies showed that hyperoxic chronic injury in newborn animals exhibits similar morphologic changes to those observed in BPD [84]. In addition to anomalous lung structure and function, infants with BPD exhibited elevated pulmonary vascular resistance and pulmonary arterial pressure [85].

The LPA_1_ pathway is regarded as a promising target for BPD therapy. Various adverse effects are mediated via LPA, such as pulmonary arterial hypertension, lung inflammation, and fibrosis [86]. In addition, Shim et al. demonstrated that rats subjected to hyperoxia exhibited significantly elevated ATX, LPA_1_, and LPA_3_ expression levels relative to rats exposed to room air. These results demonstrate that hyperoxia exposure may enhance local LPA production and contribute to BDP pathogenesis [87]. LPA-LPA_1_ signaling reduction is related to positive effects in lung illnesses, indicating the therapeutic potential of LPA_1_ occupancy. Chen and colleagues discovered that LPA_1_-deficient rats had higher BPD survival rates and were less vulnerable to a second, more severe blow, which was associated with the ERK signal transduction pathway [88]. Other studies have demonstrated that LPA_1_ deficiency decreases pulmonary injury by reducing pulmonary inflammation and fibrosis without altering alveolar and vascular development [86].

## 6. ATX/LPA Signals in Other Macrophage-Dysfunction-Related Diseases

### 6.1. Tumor

The tumor microenvironment (TME) plays a prominent role in tumor initiation, progression, and induction [89]. The tumor microenvironment is composed of macrophages, which can be categorized into three main categories: tumor-associated macrophages (TAMs) derived from monocytes, myeloid-derived suppressor cells (MDSCs), and tissue-resident macrophages. The majority of immune cells in the TME are TAMs [90].

LPA has been demonstrated to differentiate monocytes into macrophages via the Akt/mTOR pathways, with PPAR functioning as the primary regulator of this differentiation [91]. In breast cancer, LPA_3_ expression is associated with cancer-related inflammation [92]. In colorectal cancer (CRC), however, suppressing 1-acylglycerol-3-phosphate O-acyltransferase 4 (Agpat4) can stimulate the production of LPA from CRC cells, and LPA polarizes macrophages into M1-like phenotypes through LPA_1_ and LPA_3_ [93]. M1 and M2 macrophages are the two most prevalent forms of macrophages in the TME. M1 macrophages oppose tumor cells and sustain the inflammatory response by secreting nitric oxide (NO), producing pro-inflammatory cytokines, and activating immune cell responses [94]. M2 macrophages have a suppressive immune phenotype and promote tumor development [95]. TAMs can augment tumor cell migration with epidermal growth factor (EGF), proliferation with platelet-derived growth factor (PDGF), and angiogenesis with vascular endothelial growth factor (VEGF) [96,97,98].

Furthermore, LPA stimulates the production of IL-6 and IL-8 by ovarian cancer cells in the TME. IL-6 and IL-8 stimulate the differentiation of osteoclasts in vitro and recruit osteoclasts to bone metastasis sites in vivo [99]. As osteolytic bone metastasis advances, tumor cells convert LPA precursors into LPA locally by secreting ATX, and the procoagulant abilities of cancer cells are likely to release LPA and LPA precursors upon platelet aggregation [100]. LPA stimulates the secretion of IL-6 and IL-8 in oral squamous cell carcinoma (OSCC) via ERK1/2 and Akt-mediated NF-κB and AP-1 [101]. In pathological states such as bone metastasis, LPA-induced IL-6 and IL-8 increase the osteoblast receptor activator of nuclear factor (NF)-κB ligand (RANKL) expression and promote osteoblast formation from osteoblast precursors [101].

### 6.2. Atherosclerosis

Atherosclerosis is caused by the formation of atheromatous lesions on the arterial wall’s inner surface. Chronic inflammation of the arterial wall is the underlying pathology. Changes in the extracellular matrix underneath the endothelium and the endothelium’s permeability allow cholesterol-containing LDLs to enter and remain in the artery wall. Monocytes are recruited to the arterial wall at an early stage, where they consume lipoprotein particles and transform into foam cells. Smooth muscle cells (SMCs) move from the tunica media to the tunica intima at the advanced stage of atherosclerosis to create fibroatheroma plaques [102,103]. LPA and its receptors significantly promote the progression of atherosclerosis, especially in the formation of foam cells and atherosclerotic plaques. A lack of LPA_4_ reduces atherosclerosis in male mice, which correlates with an increase in M2 macrophage content. M2 macrophages tend to reduce inflammation and promote tissue repair. The transcription factor Krüppel-like factor 4 (KLF4) promotes the polarization of M2 macrophage while inhibiting M1 polarization. The absence of KLF4 hastens atherosclerosis [104]. LPA_4_ appears to inhibit the increase in KLF4 expression mediated by LPA, causing a decrease in M2 macrophage levels. Moreover, LPA_4_ regulates endothelium permeability, hematopoiesis, and lymphocyte migration, which contributes to atherosclerosis [105].

Foam cells are predominantly formed via unchecked oxidized low-density lipoprotein (ox-LDL) absorption, while excessive cholesterol esterification and hindered cholesterol release result in the accumulation of cytoplasmic lipid droplets [106]. Macrophage pattern recognition receptors are involved in the detection and internalization of ox-LDL. The two main scavenger receptors for the binding and absorption of ox-LDL are CD36 (a class B scavenger receptor) and SRA (a class A scavenger receptor) [107]. However, the reverse cholesterol transport (RCT) realized by ATP-binding cassette (ABC) transporters, such as ABCA1, ABCG2, and class B scavenger receptor type I (SRBI), mediates the excretion of intracellular unesterified cholesterol in high-density lipoproteins or apoAI [108]. LPA was shown to greatly enhance foam cell formation by disrupting the equilibrium between lipid absorption and efflux. Blocking SRBI expression via LPA_1/3_ expression and activating the AKT signaling pathway successively led to the observed findings [109]. Bioactive lipid molecules are also found to enhance the uptake of ox-LDL. LPA was shown to increase the uptake of ox-LDL in the J774 macrophage cell line [110]. In addition, repeated intravenous and intraperitoneal injection of LPS accelerates atherosclerosis in rabbits and *Apoe*^−/−^ mice [111,112]. An investigation revealed the connection between LPA and LPS in the formation of foam cells. LPA induces CD14 via LPA_1_, and CD14 facilitates the induction of scavenger receptor class A type I (SRAI) via LPA and LPS. The LPA/LPS/CD14/SRAI axis greatly improves ox-LDL absorption and stimulates the development of foam cells [113].

Besides its role in the formation of foam cells, LPA is implicated in the inflammation of atherosclerotic plaques. During the early phase of atherosclerosis, CXCL1 promotes the accumulation of macrophages and induces monocyte arrest in the vessel wall [114]. LPA accelerates the progress of atherosclerosis and recruits leukocytes to the vessel wall via the release of CXCL1 mediated by LPA_1_ and LPA_3_ [3]. NF-κB signaling and hypoxia-inducible factor (HIF)-1α are implicated in the regulation of CXCL1 expression. Unsaturated LPAs induce the upregulation of HIF-1α by activating LPA receptors in cancer cells and SMCs. HIF-1α increases microRNA-19a expression, which is associated with the activation of NF-κB, the expression of CXCL-1, and CXCL-1-dependent monocyte adhesion [115]. According to another study, lipoprotein-associated phospholipase A_2_ (Lp-PLA_2_) hydrolyses oxidized the phospholipid in LDL to produce lysophosphatidylcholine (lysoPC), which plays a crucial role in the inflammation of human atherosclerotic plaques. This observation indicates that inhibiting Lp-PLA_2_ is a promising therapeutic strategy. Moreover, the relationship between lysoPC and plaque inflammation may result from LPA rather than being a direct effect of lysoPC [116]. MMPs are zinc-dependent enzymes and participate in the degradation and remodeling of the extracellular matrix. MMPs may accelerate plaque disruption [117]. Particularly, MMP-9 content is associated with plaque stability. Fan et al. revealed that MMP-9 derived from macrophages was related to coronary plaque instability [118]. LPA activates the NF-κB signaling pathway to promote the expression and bioactivity of MMP-9 via LPA_2_ [119]. Mast cell activation has a crucial role in the development and destabilization of plaque. LPA levels rise in or near the plaque as it progresses, increasing the number of macrophages and causing vascular leakage by activating mast cells. A fraction of hematopoietic cells and potentially harmful substances enter atherosclerotic plaques because of microvascular leakage, which destabilizes the plaques [120].

LPA content regulation is another factor that regulates atherosclerosis. LPA availability is regulated by lipid phosphate phosphatase 3 (LPP3), which is encoded by the PLPP3 gene. LPP3 expression in SMCs controls LPA-induced Rho activity, ERK activation, and migration. Consequently, SMC LPP3 is a crucial factor in the progression of atherosclerosis and LPA content in lesions [121]. LPA_6_ expressed by endothelial cells is the most prevalent LPA receptor in plaques and positively correlates with PLPP3 gene expression. LPA_2_ and LPA_5_ are also upregulated in carotid atherosclerotic lesions, while LPA_1_ is the only LPA receptor that is downregulated [122].

### 6.3. Fibrosis

The ATX/LPA signaling axis is apparent in pulmonary fibrosis. ATX is predominantly expressed in alveolar macrophages and bronchial epithelial cells. The conditional deletion of *ENPP2* in both pulmonary cell compartments diminishes lung fibrosis, implicating ATX in the pathogenesis of the disease [123]. Peroxiredoxin 6 (Prdx6)-LPA_2_ signaling transduction modulates NADPH oxidase 2 (NOX2) activation in alveolar macrophages (AMs) and pulmonary microvascular endothelial cells (PMVECs) [124,125]. The single-cell RNA sequence data of ATX-expressing cells revealed these two macrophage populations, including resident macrophages (MRes-FABP4+) and monocyte-derived macrophages (MDM-FCN1+), in the bronchoalveolar lavage (BAL) fluid of a lung transplant recipient with chronic lung allograft dysfunction (CLAD). MDM exhibited proinflammatory properties and generated the highest ATX levels, suggesting that it may be the initiator of the ATX-LPA cascade. LPA was also shown to initiate MSC migration and fibrotic contraction, indicating that ATX-induced LPA is central to the pathogenesis of CLAD [126]. ATX-expressing alveolar macrophages have also been detected in the BAL fluid of CLAD patients. These cells may be the source of ATX/PLA, which drives mesenchymal stem cell aggregation and tissue contraction. This finding suggests that CLAD and another form of pulmonary fibrosis share a common pathogenesis [126].

Other research on idiopathic pulmonary fibrosis (IPF) revealed that the inhibition of LPA_1_ reduced fibroblasts’ responses to chemotactic stimuli [127]. In addition, LPA signaling via LPA_1_ induced the apoptosis of normal bronchial epithelial cells while promoting lung fibroblasts’ resistance to apoptosis [128]. Therefore, LPA signaling promotes lung fibrosis via LPA_1_. TGF-β is a fibrotic factor prototype with effects on alveolar epithelial cell injury, extracellular matrix regulation and remodeling, myofibroblast differentiation, and epithelial–mesenchymal transition (EMT) [129]. Mammalian TGF-β exists in three isoforms: TGF-β1, TGF-β2, and TGF-β3. These TGF-β isoforms possess similar biofunctions and significantly regulate lung development, inflammation, repair, and injury. Consequently, TGF-β activation is an additional important mechanism in fibrosis, and TGF-β is a promising therapeutic target for pulmonary fibrosis [130]. LPA_2_ deletion attenuated the apoptosis of alveolar and bronchial epithelial cells in the murine lung. In general, LPA_2_ deficiency reduces lung injury and lung fibrosis [131]. The role of LPA_3–6_ in IPF and lung fibrosis remains to be discovered.

## 7. Intervention Strategies Targeting LPA Metabolism in Macrophages and Diseases

Due to LPA’s critical functions in a range of pathogenic events mediated by macrophages, numerous researchers have investigated intervention tactics targeting its metabolism. In general, the approaches concentrate on blocking LPA receptors and reducing ATX.

### 7.1. Intervention Strategies in LPA Receptors

LPA has been found to target LPA_1_ and cause neuron apoptosis. Preclinical research has suggested a connection between depression and hippocampal neuronal death [132]. According to Xu et al., Saikosaponin D reduced depressive-like behaviors caused by LPS via control of the LPA_1_/RhoA/ROCK2 signaling pathway, which, in turn, prevented neuronal death [133]. LPA synthesis and signaling transduction are also connected to the start of neuronal pain (NP). Numerous studies have shown that LPA_1_ and LPA_5_ can activate NP via various pathways [134,135]. Therefore, limiting LPA_1_ and/or LPA_5_ downstream signaling cascades or focusing on LPA synthesis by inhibiting ATX could be potential methods for preventing the development of NP [136]. C22:5 and C22:6 polyunsaturated LPAs can potentially serve as asthma markers [137]. Additionally, the discovery of asthma biomarkers may be used for patient-specific care and treatment, disease diagnosis, and disease severity. Moreover, LPA generated from activated blood platelets [4] works as a tumor mitogen and inducer of tumor cytokines and is absorbed by breast and ovarian cancers, hence promoting the progression of bone metastases [99]. Further evidence that LPA_1_ is important for immunological infiltrates in prostate cancer and may serve as a therapeutic target derives from its association with immune cell migration in this disease [138].

In clinical trials, LPA_1_ inhibitors are mainly used in intervention strategies for IPF and systemic sclerosis (Table 2). In detail, the ability of LPA_1_ antagonism to promote antifibrosis in lung fibrosis greatly impacts ECM remodeling and lung function in IPF patients [139]. Regarding systemic sclerosis, LPA_1_ inhibition reduces or even reverses the progression of fibrosis [140]. LPA_3_ is the sole LPA receptor that is differentially expressed in ovarian cancer, and LPA elevates the migration and proliferation of LPA_3_-overexpressing cancer cells. The data suggest that LPA may be an ideal therapeutic target for ovarian cancer [141].

### 7.2. Invention Strategies for the Attenuation of ATX

By increasing LPA synthesis, ATX may diminish the efficacy of cancer therapy. In breast cancer treatment, the ATX inhibitor GLPG1690 reduces the concentration of LPA in the tumor microenvironment, hence enhancing the efficacy of conventional chemotherapy and radiotherapy. Furthermore, GLPG1690 may theoretically inhibit the development of radiation-induced fibrosis [142]. In clinical trials, ATX inhibitors are mainly used in intervention strategies for IPF, metastatic pancreatic cancer, and chronic liver disease (Table 2).

The ATX-LPA axis is also essential for the development of cardiovascular disease. LPA impacts the behavior of blood cells and vascular cells via the transduction of downstream signaling. Specifically, LPA stimulates the production of thrombus upon an atherosclerotic plaque’s rupture via LPA_5_ [143], increases the migration of endothelial cells [144], and induces monocyte differentiation into macrophages [91] and foam cell formation via LPA_1–3_ [109]. The use of ATX inhibitors such as GLPG1690 or monoclonal antibodies is being evaluated to decrease LPA. Although the majority of these medication candidates are not intended specifically for cardiovascular conditions, their potential to reduce circulating LPA levels remains promising [145].

## 8. Conclusions

Many discoveries indicate that LPA and its receptor signaling display multiple effects in macrophage dysfunction and inflammation-related diseases, including autoimmune encephalomyelitis, IBD, asthma, RA, BPD, tumors, atherosclerosis, and fibrosis. However, the exact molecular mechanism is largely unknown, and these findings on the roles and intervention value of LPA metabolism and/or signals are mainly based on data from in vitro and or in vivo mouse models. Additionally, there remains the need for large-sample-sized clinical studies to illustrate and evaluate the effects and safety of LPA receptor antagonists and ATX inhibitors in the treatment of macrophage-dysfunction- and inflammation-related diseases.

## Figures and Tables

**Table 1:** LPA receptors: intercellular functions.

Receptors	G Protein	Cascade Pathways	Functions	Reference
LPA_1_/Edg2/vzg-1	G_α12/13_, G_αq/11_, and G_αi_	PLC, MAPK, Akt, Rho, and YAP/Taz activation	Cerebral cortex formation and function; Myelination; Astrocyte proliferation and astrogliosis; Cell proliferation; SRE activation; Activation of AC inhibition; Ca^2+^ mobilization; Development of neuropathic pain.	[11,12,13,14]
LPA_2_/Edg4	G_α12/13_, G_αq/11_, and G_αi_	MAPK, PLC, Akt, Notch, and Rho activation	AC inhibition; Ca^2+^ mobilization; SRE activation; Neurogenesis; Ovarian cancer aggressiveness; Migration and invasion activities of SGc-7901 gastric cancer cells.	[15,16,17]
LPA_3_/Edg7	G_αq_/G_αi_	PLC, YAP/Taz, and MAPK activation	Ca^2+^ mobilization; AC inhibition and activation; Embryo implantation and altering embryo spacing; Ovarian cancer aggressiveness.	[16,18,19]
LPA_4_/p2y9/GPR23	G_α12/13_, G_αq/11_, G_αi_, and G_αs_	Rho/ROCK, MAPK, Akt, and PLC activation	Cell aggregation; N-cadherin-dependent cell adhesion; Intracellular cAMP accumulation; Negatively regulates cell motility.	[8,20,21,22,23]
LPA_5_/GPR92	G_α12/13_ and G_αq/11_	PLC activation	Neurite retraction and stress fiber formation; Increases cAMP levels and inositol phosphate production; Affects water absorption in the colon; Ca^2+^ mobilization.	[8,24,25]
LPA_6_/p2y5	G_12/13_	Rho activation	AC activation; Neurite retraction in B103-LPA_6_ cells; Membrane blebbing in RH7777-LPA_6_ cells; Involved in hypotrichosis simplex; Involved in metastasis of androgen-independent prostate cancer cells.	[26,27,28]

Abbreviations: PLC—phospholipase C; MAPK—mitogen-activated protein kinase; Edg—endothelial differentiating gene; YAP—yes-associated protein; ROCK—Rho-associated protein kinase; SRE—serum-responsive element; AC—adenylyl cyclase; cAMP—cyclic adenosine 3,5-monophosphate.

**Table 2:** Clinical trials using intervention strategies targeting LPA metabolism.

Disease	Target	Drug Name	Phase	ClinicalTrials.gov Identifier
Idiopathic pulmonary fibrosis	LPA_1_ inhibitor	BMS-986020	2	NCT01766817
Idiopathic pulmonary fibrosis	LPA_1_ inhibitor	18F-BMS-986327	1	NCT04069143
Idiopathic pulmonary fibrosis	ATX inhibitor	BBT-877	1	NCT03830125
Idiopathic pulmonary fibrosis	ATX inhibitor	GLPG1690	2	NCT02738801
Metastatic pancreatic cancer	ATX inhibitor	IOA-289	1/2	NCT05586516
Chronic liver disease	ATX inhibitor	BLD-0409	1	NCT04146805
Systemic sclerosis	LPA_1_ inhibitor	SAR100842	2	NCT01651143

Data extracted from www.clinicaltrials.gov (accessed on 19 June 2023).

## Data Availability

No data were used for the research described in this article.

## References

[1] Wynn T.A., Chawla A., Pollard J.W. (2013). Macrophage biology in development, homeostasis and disease. Nature.

[2] Yung Y.C., Stoddard N.C., Chun J. (2014). LPA receptor signaling: Pharmacology, physiology, and pathophysiology. J. Lipid Res..

[3] Zhou Z., Subramanian P., Sevilmis G., Globke B., Soehnlein O., Karshovska E., Megens R., Heyll K., Chun J., Saulnier-Blache J.S. (2011). Lipoprotein-derived lysophosphatidic acid promotes atherosclerosis by releasing CXCL1 from the endothelium. Cell Metab..

[4] Aoki J., Taira A., Takanezawa Y., Kishi Y., Hama K., Kishimoto T., Mizuno K., Saku K., Taguchi R., Arai H. (2002). Serum lysophosphatidic acid is produced through diverse phospholipase pathways. J. Biol. Chem..

[5] Aoki J., Inoue A., Okudaira S. (2008). Two pathways for lysophosphatidic acid production. Biochim. Biophys. Acta.

[6] Stefan C., Jansen S., Bollen M. (2005). NPP-type ectophosphodiesterases: Unity in diversity. Trends Biochem. Sci..

[7] van Meeteren L.A., Ruurs P., Stortelers C., Bouwman P., van Rooijen M.A., Pradère J.P., Pettit T.R., Wakelam M.J., Saulnier-Blache J.S., Mummery C.L. (2006). Autotaxin, a secreted lysophospholipase D, is essential for blood vessel formation during development. Mol. Cell Biol..

[8] Geraldo L.H.M., Spohr T., Amaral R.F.D., Fonseca A., Garcia C., Mendes F.A., Freitas C., dosSantos M.F., Lima F.R.S. (2021). Role of lysophosphatidic acid and its receptors in health and disease: Novel therapeutic strategies. Signal Transduct. Target. Ther..

[9] Brindley D.N., Pilquil C. (2009). Lipid phosphate phosphatases and signaling. J. Lipid Res..

[10] Vancura A., Carroll M.A., Haldar D. (1991). A lysophosphatidic acid-binding cytosolic protein stimulates mitochondrial glycerophosphate acyltransferase. Biochem. Biophys. Res. Commun..

[11] Hecht J.H., Weiner J.A., Post S.R., Chun J. (1996). Ventricular zone gene-1 (vzg-1) encodes a lysophosphatidic acid receptor expressed in neurogenic regions of the developing cerebral cortex. J. Cell Biol..

[12] Sorensen S.D., Nicole O., Peavy R.D., Montoya L.M., Lee C.J., Murphy T.J., Traynelis S.F., Hepler J.R. (2003). Common signaling pathways link activation of murine PAR-1, LPA, and S1P receptors to proliferation of astrocytes. Mol. Pharmacol..

[13] Ishii I., Fukushima N., Ye X., Chun J. (2004). Lysophospholipid receptors: Signaling and biology. Annu. Rev. Biochem..

[14] Inoue M., Rashid M.H., Fujita R., Contos J.J., Chun J., Ueda H. (2004). Initiation of neuropathic pain requires lysophosphatidic acid receptor signaling. Nat. Med..

[15] Ren Z., Zhang C., Ma L., Zhang X., Shi S., Tang D., Xu J., Hu Y., Wang B., Zhang F. (2019). Lysophosphatidic acid induces the migration and invasion of SGC-7901 gastric cancer cells through the LPA2 and Notch signaling pathways. Int. J. Mol. Med..

[16] Yu S., Murph M.M., Lu Y., Liu S., Hall H.S., Liu J., Stephens C., Fang X., Mills G.B. (2008). Lysophosphatidic acid receptors determine tumorigenicity and aggressiveness of ovarian cancer cells. J. Natl. Cancer Inst..

[17] Fukushima N., Ishii I., Contos J.J., Weiner J.A., Chun J. (2001). Lysophospholipid receptors. Annu. Rev. Pharmacol. Toxicol..

[18] Ye X., Hama K., Contos J.J., Anliker B., Inoue A., Skinner M.K., Suzuki H., Amano T., Kennedy G., Arai H. (2005). LPA3-mediated lysophosphatidic acid signalling in embryo implantation and spacing. Nature.

[19] Ishii I., Contos J.J., Fukushima N., Chun J. (2000). Functional comparisons of the lysophosphatidic acid receptors, LP(A1)/VZG-1/EDG-2, LP(A2)/EDG-4, and LP(A3)/EDG-7 in neuronal cell lines using a retrovirus expression system. Mol. Pharmacol..

[20] Lee C.W., Rivera R., Dubin A.E., Chun J. (2007). LPA(4)/GPR23 is a lysophosphatidic acid (LPA) receptor utilizing G(s)-, G(q)/G(i)-mediated calcium signaling and G(12/13)-mediated Rho activation. J. Biol. Chem..

[21] Yanagida K., Ishii S., Hamano F., Noguchi K., Shimizu T. (2007). LPA4/p2y9/GPR23 mediates rho-dependent morphological changes in a rat neuronal cell line. J. Biol. Chem..

[22] Lee Z., Cheng C.T., Zhang H., Subler M.A., Wu J., Mukherjee A., Windle J.J., Chen C.K., Fang X. (2008). Role of LPA4/p2y9/GPR23 in negative regulation of cell motility. Mol. Biol. Cell.

[23] Noguchi K., Ishii S., Shimizu T. (2003). Identification of p2y9/GPR23 as a novel G protein-coupled receptor for lysophosphatidic acid, structurally distant from the Edg family. J. Biol. Chem..

[24] Lee C.W., Rivera R., Gardell S., Dubin A.E., Chun J. (2006). GPR92 as a new G12/13- and Gq-coupled lysophosphatidic acid receptor that increases cAMP, LPA5. J. Biol. Chem..

[25] Lin S., Yeruva S., He P., Singh A.K., Zhang H., Chen M., Lamprecht G., de Jonge H.R., Tse M., Donowitz M. (2010). Lysophosphatidic acid stimulates the intestinal brush border Na(+)/H(+) exchanger 3 and fluid absorption via LPA(5) and NHERF2. Gastroenterology.

[26] Yanagida K., Masago K., Nakanishi H., Kihara Y., Hamano F., Tajima Y., Taguchi R., Shimizu T., Ishii S. (2009). Identification and characterization of a novel lysophosphatidic acid receptor, p2y5/LPA6. J. Biol. Chem..

[27] Pasternack S.M., von Kügelgen I., Al Aboud K., Lee Y.A., Rüschendorf F., Voss K., Hillmer A.M., Molderings G.J., Franz T., Ramirez A. (2008). G protein-coupled receptor P2Y5 and its ligand LPA are involved in maintenance of human hair growth. Nat. Genet..

[28] Ketscher A., Jilg C.A., Willmann D., Hummel B., Imhof A., Rüsseler V., Hölz S., Metzger E., Müller J.M., Schüle R. (2014). LSD1 controls metastasis of androgen-independent prostate cancer cells through PXN and LPAR6. Oncogenesis.

[29] Walker T.L., Overall R.W., Vogler S., Sykes A.M., Ruhwald S., Lasse D., Ichwan M., Fabel K., Kempermann G. (2016). Lysophosphatidic Acid Receptor Is a Functional Marker of Adult Hippocampal Precursor Cells. Stem Cell Rep..

[30] Park J., Jang J.H., Oh S., Kim M., Shin C., Jeong M., Heo K., Park J.B., Kim S.R., Oh Y.S. (2018). LPA-induced migration of ovarian cancer cells requires activation of ERM proteins via LPA(1) and LPA(2). Cell. Signal..

[31] Bandoh K., Aoki J., Hosono H., Kobayashi S., Kobayashi T., Murakami-Murofushi K., Tsujimoto M., Arai H., Inoue K. (1999). Molecular cloning and characterization of a novel human G-protein-coupled receptor, EDG7, for lysophosphatidic acid. J. Biol. Chem..

[32] Ohuchi H., Hamada A., Matsuda H., Takagi A., Tanaka M., Aoki J., Arai H., Noji S. (2008). Expression patterns of the lysophospholipid receptor genes during mouse early development. Dev. Dyn..

[33] Taniguchi R., Inoue A., Sayama M., Uwamizu A., Yamashita K., Hirata K., Yoshida M., Tanaka Y., Kato H.E., Nakada-Nakura Y. (2017). Structural insights into ligand recognition by the lysophosphatidic acid receptor LPA(6). Nature.

[34] Issemann I., Green S. (1990). Activation of a member of the steroid hormone receptor superfamily by peroxisome proliferators. Nature.

[35] Ricote M., Huang J., Fajas L., Li A., Welch J., Najib J., Witztum J.L., Auwerx J., Palinski W., Glass C.K. (1998). Expression of the peroxisome proliferator-activated receptor gamma (PPARgamma) in human atherosclerosis and regulation in macrophages by colony stimulating factors and oxidized low density lipoprotein. Proc. Natl. Acad. Sci. USA.

[36] Duong C.Q., Bared S.M., Abu-Khader A., Buechler C., Schmitz A., Schmitz G. (2004). Expression of the lysophospholipid receptor family and investigation of lysophospholipid-mediated responses in human macrophages. Biochim. Biophys. Acta.

[37] Koh J.S., Lieberthal W., Heydrick S., Levine J.S. (1998). Lysophosphatidic acid is a major serum noncytokine survival factor for murine macrophages which acts via the phosphatidylinositol 3-kinase signaling pathway. J. Clin. Investig..

[38] Worthylake R.A., Lemoine S., Watson J.M., Burridge K. (2001). RhoA is required for monocyte tail retraction during transendothelial migration. J. Cell Biol..

[39] Davies M.R., Lee L., Feeley B.T., Kim H.T., Liu X. (2017). Lysophosphatidic acid-induced RhoA signaling and prolonged macrophage infiltration worsens fibrosis and fatty infiltration following rotator cuff tears. J. Orthop. Res..

[40] Moore K.J., Sheedy F.J., Fisher E.A. (2013). Macrophages in atherosclerosis: A dynamic balance. Nat. Rev. Immunol..

[41] Llodrá J., Angeli V., Liu J., Trogan E., Fisher E.A., Randolph G.J. (2004). Emigration of monocyte-derived cells from atherosclerotic lesions characterizes regressive, but not progressive, plaques. Proc. Natl. Acad. Sci. USA.

[42] Chen L., Zhang J., Yang X., Liu Y., Deng X., Yu C. (2019). Lysophosphatidic acid decreased macrophage foam cell migration correlated with downregulation of fucosyltransferase 8 via HNF1α. Atherosclerosis.

[43] Colonna M., Butovsky O. (2017). Microglia Function in the Central Nervous System During Health and Neurodegeneration. Annu. Rev. Immunol..

[44] Amaral R.F., Geraldo L.H.M., Einicker-Lamas M., TCLS E.S., Mendes F., Lima F.R.S. (2021). Microglial lysophosphatidic acid promotes glioblastoma proliferation and migration via LPA(1) receptor. J. Neurochem..

[45] Kim K.S., Sengupta S., Berk M., Kwak Y.G., Escobar P.F., Belinson J., Mok S.C., Xu Y. (2006). Hypoxia enhances lysophosphatidic acid responsiveness in ovarian cancer cells and lysophosphatidic acid induces ovarian tumor metastasis in vivo. Cancer Res..

[46] Bar E.E. (2011). Glioblastoma, cancer stem cells and hypoxia. Brain Pathol..

[47] Krishnamoorthy S., Honn K.V. (2006). Inflammation and disease progression. Cancer Metastasis Rev..

[48] Benesch M.G., Ko Y.M., McMullen T.P., Brindley D.N. (2014). Autotaxin in the crosshairs: Taking aim at cancer and other inflammatory conditions. FEBS Lett..

[49] Benesch M.G.K., MacIntyre I.T.K., McMullen T.P.W., Brindley D.N. (2018). Coming of Age for Autotaxin and Lysophosphatidate Signaling: Clinical Applications for Preventing, Detecting and Targeting Tumor-Promoting Inflammation. Cancers.

[50] Tang X., Wang X., Zhao Y.Y., Curtis J.M., Brindley D.N. (2017). Doxycycline attenuates breast cancer related inflammation by decreasing plasma lysophosphatidate concentrations and inhibiting NF-κB activation. Mol. Cancer.

[51] Chang C.L., Lin M.E., Hsu H.Y., Yao C.L., Hwang S.M., Pan C.Y., Hsu C.Y., Lee H. (2008). Lysophosphatidic acid-induced interleukin-1 beta expression is mediated through Gi/Rho and the generation of reactive oxygen species in macrophages. J. Biomed. Sci..

[52] Gaire B.P., Lee C.H., Kim W., Sapkota A., Lee D.Y., Choi J.W. (2020). Lysophosphatidic Acid Receptor 5 Contributes to Imiquimod-Induced Psoriasis-Like Lesions through NLRP3 Inflammasome Activation in Macrophages. Cells.

[53] Lee C.H., Sapkota A., Gaire B.P., Choi J.W. (2020). NLRP3 Inflammasome Activation Is Involved in LPA(1)-Mediated Brain Injury after Transient Focal Cerebral Ischemia. Int. J. Mol. Sci..

[54] Mirzoyan K., Denis C., Casemayou A., Gilet M., Marsal D., Goudounéche D., Faguer S., Bascands J.L., Schanstra J.P., Saulnier-Blache J.S. (2017). Lysophosphatidic Acid Protects Against Endotoxin-Induced Acute Kidney Injury. Inflammation.

[55] Chien H.Y., Lu C.S., Chuang K.H., Kao P.H., Wu Y.L. (2015). Attenuation of LPS-induced cyclooxygenase-2 and inducible NO synthase expression by lysophosphatidic acid in macrophages. Innate Immun..

[56] Ciesielska A., Hromada-Judycka A., Ziemlińska E., Kwiatkowska K. (2019). Lysophosphatidic acid up-regulates IL-10 production to inhibit TNF-α synthesis in Mϕs stimulated with LPS. J. Leukoc. Biol..

[57] Reich D.S., Lucchinetti C.F., Calabresi P.A. (2018). Multiple Sclerosis. N. Engl. J. Med..

[58] Constantinescu C.S., Farooqi N., O’Brien K., Gran B. (2011). Experimental autoimmune encephalomyelitis (EAE) as a model for multiple sclerosis (MS). Br. J. Pharmacol..

[59] Ninou I., Sevastou I., Magkrioti C., Kaffe E., Stamatakis G., Thivaios S., Panayotou G., Aoki J., Kollias G., Aidinis V. (2020). Genetic deletion of Autotaxin from CD11b+ cells decreases the severity of experimental autoimmune encephalomyelitis. PLoS ONE.

[60] Fransson J., Gómez-Conde A.I., Romero-Imbroda J., Fernández O., Leyva L., de Fonseca F.R., Chun J., Louapre C., Van-Evercooren A.B., Zujovic V. (2021). Activation of Macrophages by Lysophosphatidic Acid through the Lysophosphatidic Acid Receptor 1 as a Novel Mechanism in Multiple Sclerosis Pathogenesis. Mol. Neurobiol..

[61] Bain C.C., Schridde A. (2018). Origin, Differentiation, and Function of Intestinal Macrophages. Front. Immunol..

[62] Hozumi H., Hokari R., Kurihara C., Narimatsu K., Sato H., Sato S., Ueda T., Higashiyama M., Okada Y., Watanabe C. (2013). Involvement of autotaxin/lysophospholipase D expression in intestinal vessels in aggravation of intestinal damage through lymphocyte migration. Lab. Investig..

[63] Wang Z., Shi W., Tian D., Qin H., Vallance B.A., Yang H., Yu H.B., Yu Q. (2020). Autotaxin stimulates LPA2 receptor in macrophages and exacerbates dextran sulfate sodium-induced acute colitis. J. Mol. Med..

[64] Kim S.J., Howe C., Mitchell J., Choo J., Powers A., Oikonomopoulos A., Pothoulakis C., Hommes D.W., Im E., Rhee S.H. (2020). Autotaxin loss accelerates intestinal inflammation by suppressing TLR4-mediated immune responses. EMBO Rep..

[65] Kaufman G. (2011). Asthma: Pathophysiology, diagnosis and management. Nurs. Stand..

[66] Gauvreau G.M., Davis B.E., Scadding G., Boulet L.P., Bjermer L., Chaker A., Cockcroft D.W., Dahlén B., Fokkens W., Hellings P. (2022). Allergen provocation tests in respiratory research: Building on 50 years of experience. Eur. Respir. J..

[67] Georas S.N., Berdyshev E., Hubbard W., Gorshkova I.A., Usatyuk P.V., Saatian B., Myers A.C., Williams M.A., Xiao H.Q., Liu M. (2007). Lysophosphatidic acid is detectable in human bronchoalveolar lavage fluids at baseline and increased after segmental allergen challenge. Clin. Exp. Allergy.

[68] Park G.Y., Lee Y.G., Berdyshev E., Nyenhuis S., Du J., Fu P., Gorshkova I.A., Li Y., Chung S., Karpurapu M. (2013). Autotaxin production of lysophosphatidic acid mediates allergic asthmatic inflammation. Am. J. Respir. Crit. Care Med..

[69] Rubenfeld J., Guo J., Sookrung N., Chen R., Chaicumpa W., Casolaro V., Zhao Y., Natarajan V., Georas S. (2006). Lysophosphatidic acid enhances interleukin-13 gene expression and promoter activity in T cells. Am. J. Physiol. Lung Cell Mol. Physiol..

[70] Idzko M., Laut M., Panther E., Sorichter S., Dürk T., Fluhr J.W., Herouy Y., Mockenhaupt M., Myrtek D., Elsner P. (2004). Lysophosphatidic acid induces chemotaxis, oxygen radical production, CD11b up-regulation, Ca2+ mobilization, and actin reorganization in human eosinophils via pertussis toxin-sensitive G proteins. J. Immunol..

[71] Lee Y.J., Im D.S. (2022). Efficacy Comparison of LPA(2) Antagonist H2L5186303 and Agonist GRI977143 on Ovalbumin-Induced Allergic Asthma in BALB/c Mice. Int. J. Mol. Sci..

[72] Emo J., Meednu N., Chapman T.J., Rezaee F., Balys M., Randall T., Rangasamy T., Georas S.N. (2012). Lpa2 is a negative regulator of both dendritic cell activation and murine models of allergic lung inflammation. J. Immunol..

[73] Kim S.J., Moon H.G., Park G.Y. (2020). The roles of autotaxin/lysophosphatidic acid in immune regulation and asthma. Biochim. Biophys. Acta Mol. Cell Biol. Lipids.

[74] McInnes I.B., Schett G. (2011). The pathogenesis of rheumatoid arthritis. N. Engl. J. Med..

[75] Müller-Ladner U., Ospelt C., Gay S., Distler O., Pap T. (2007). Cells of the synovium in rheumatoid arthritis. Synovial fibroblasts. Arthritis Res. Ther..

[76] Cejka D., Hayer S., Niederreiter B., Sieghart W., Fuereder T., Zwerina J., Schett G. (2010). Mammalian target of rapamycin signaling is crucial for joint destruction in experimental arthritis and is activated in osteoclasts from patients with rheumatoid arthritis. Arthritis Rheum..

[77] Walsh N.C., Gravallese E.M. (2010). Bone remodeling in rheumatic disease: A question of balance. Immunol. Rev..

[78] Nochi H., Tomura H., Tobo M., Tanaka N., Sato K., Shinozaki T., Kobayashi T., Takagishi K., Ohta H., Okajima F. (2008). Stimulatory role of lysophosphatidic acid in cyclooxygenase-2 induction by synovial fluid of patients with rheumatoid arthritis in fibroblast-like synovial cells. J. Immunol..

[79] Zhao C., Fernandes M.J., Prestwich G.D., Turgeon M., Di Battista J., Clair T., Poubelle P.E., Bourgoin S.G. (2008). Regulation of lysophosphatidic acid receptor expression and function in human synoviocytes: Implications for rheumatoid arthritis?. Mol. Pharmacol..

[80] Nikitopoulou I., Oikonomou N., Karouzakis E., Sevastou I., Nikolaidou-Katsaridou N., Zhao Z., Mersinias V., Armaka M., Xu Y., Masu M. (2012). Autotaxin expression from synovial fibroblasts is essential for the pathogenesis of modeled arthritis. J. Exp. Med..

[81] Orosa B., García S., Martínez P., González A., Gómez-Reino J.J., Conde C. (2014). Lysophosphatidic acid receptor inhibition as a new multipronged treatment for rheumatoid arthritis. Ann. Rheum. Dis..

[82] Shen P., Jiao Y., Miao L., Chen J.H., Momtazi-Borojeni A.A. (2020). Immunomodulatory effects of berberine on the inflamed joint reveal new therapeutic targets for rheumatoid arthritis management. J. Cell Mol. Med..

[83] Mosca F., Colnaghi M., Fumagalli M. (2011). BPD: Old and new problems. J. Matern. Fetal Neonatal Med..

[84] Bhandari V. (2010). Hyperoxia-derived lung damage in preterm infants. Semin. Fetal Neonatal Med..

[85] Ambalavanan N., Mourani P. (2014). Pulmonary hypertension in bronchopulmonary dysplasia. Birth Defects Res. A Clin. Mol. Teratol..

[86] Chen X., Walther F.J., van Boxtel R., Laghmani E.H., Sengers R.M., Folkerts G., DeRuiter M.C., Cuppen E., Wagenaar G.T. (2016). Deficiency or inhibition of lysophosphatidic acid receptor 1 protects against hyperoxia-induced lung injury in neonatal rats. Acta Physiol..

[87] Shim G.H., Kim H.S., Kim E.S., Lee K.Y., Kim E.K., Choi J.H. (2015). Expression of autotaxin and lysophosphatidic acid receptors 1 and 3 in the developing rat lung and in response to hyperoxia. Free Radic. Res..

[88] Chen X., Walther F.J., Laghmani E.H., Hoogeboom A.M., Hogen-Esch A.C., van Ark I., Folkerts G., Wagenaar G.T. (2017). Adult Lysophosphatidic Acid Receptor 1-Deficient Rats with Hyperoxia-Induced Neonatal Chronic Lung Disease Are Protected against Lipopolysaccharide-Induced Acute Lung Injury. Front. Physiol..

[89] Bissell M.J., Hines W.C. (2011). Why don’t we get more cancer? A proposed role of the microenvironment in restraining cancer progression. Nat. Med..

[90] Dehne N., Mora J., Namgaladze D., Weigert A., Brüne B. (2017). Cancer cell and macrophage cross-talk in the tumor microenvironment. Curr. Opin. Pharmacol..

[91] Ray R., Rai V. (2017). Lysophosphatidic acid converts monocytes into macrophages in both mice and humans. Blood.

[92] Cha Y.J., Koo J.S. (2019). Expression of Autotaxin-Lysophosphatidate Signaling-Related Proteins in Breast Cancer with Adipose Stroma. Int. J. Mol. Sci..

[93] Zhang D., Shi R., Xiang W., Kang X., Tang B., Li C., Gao L., Zhang X., Zhang L., Dai R. (2020). The Agpat4/LPA axis in colorectal cancer cells regulates antitumor responses via p38/p65 signaling in macrophages. Signal Transduct. Target. Ther..

[94] Boutilier A.J., Elsawa S.F. (2021). Macrophage Polarization States in the Tumor Microenvironment. Int. J. Mol. Sci..

[95] Biswas S.K., Mantovani A. (2010). Macrophage plasticity and interaction with lymphocyte subsets: Cancer as a paradigm. Nat. Immunol..

[96] Wyckoff J., Wang W., Lin E.Y., Wang Y., Pixley F., Stanley E.R., Graf T., Pollard J.W., Segall J., Condeelis J. (2004). A paracrine loop between tumor cells and macrophages is required for tumor cell migration in mammary tumors. Cancer Res..

[97] Vignaud J.M., Marie B., Klein N., Plénat F., Pech M., Borrelly J., Martinet N., Duprez A., Martinet Y. (1994). The role of platelet-derived growth factor production by tumor-associated macrophages in tumor stroma formation in lung cancer. Cancer Res..

[98] Lewis J.S., Landers R.J., Underwood J.C., Harris A.L., Lewis C.E. (2000). Expression of vascular endothelial growth factor by macrophages is up-regulated in poorly vascularized areas of breast carcinomas. J. Pathol..

[99] Boucharaba A., Serre C.M., Grès S., Saulnier-Blache J.S., Bordet J.C., Guglielmi J., Clézardin P., Peyruchaud O. (2004). Platelet-derived lysophosphatidic acid supports the progression of osteolytic bone metastases in breast cancer. J. Clin. Investig..

[100] David M., Wannecq E., Descotes F., Jansen S., Deux B., Ribeiro J., Serre C.M., Grès S., Bendriss-Vermare N., Bollen M. (2010). Cancer cell expression of autotaxin controls bone metastasis formation in mouse through lysophosphatidic acid-dependent activation of osteoclasts. PLoS ONE.

[101] Hwang Y.S., Lee S.K., Park K.-K., Chung W.-Y. (2012). Secretion of IL-6 and IL-8 from lysophosphatidic acid-stimulated oral squamous cell carcinoma promotes osteoclastogenesis and bone resorption. Oral. Oncol..

[102] Weber C., Noels H. (2011). Atherosclerosis: Current pathogenesis and therapeutic options. Nat. Med..

[103] Libby P., Ridker P.M., Hansson G.K. (2011). Progress and challenges in translating the biology of atherosclerosis. Nature.

[104] Liao X., Sharma N., Kapadia F., Zhou G., Lu Y., Hong H., Paruchuri K., Mahabeleshwar G.H., Dalmas E., Venteclef N. (2011). Krüppel-like factor 4 regulates macrophage polarization. J. Clin. Investig..

[105] Yang L., Kraemer M., Fang X.F., Angel P.M., Drake R.R., Morris A.J., Smyth S.S. (2019). LPA receptor 4 deficiency attenuates experimental atherosclerosis. J. Lipid Res..

[106] Seo H.S., Choi M.H. (2015). Cholesterol homeostasis in cardiovascular disease and recent advances in measuring cholesterol signatures. J. Steroid Biochem. Mol. Biol..

[107] Kunjathoor V.V., Febbraio M., Podrez E.A., Moore K.J., Andersson L., Koehn S., Rhee J.S., Silverstein R., Hoff H.F., Freeman M.W. (2002). Scavenger receptors class A-I/II and CD36 are the principal receptors responsible for the uptake of modified low density lipoprotein leading to lipid loading in macrophages. J. Biol. Chem..

[108] Phillips M.C. (2014). Molecular mechanisms of cellular cholesterol efflux. J. Biol. Chem..

[109] Chen L., Zhang J., Deng X., Liu Y., Yang X., Wu Q., Yu C. (2017). Lysophosphatidic acid directly induces macrophage-derived foam cell formation by blocking the expression of SRBI. Biochem. Biophys. Res. Commun..

[110] Chang C.L., Hsu H.Y., Lin H.Y., Chiang W., Lee H. (2008). Lysophosphatidic acid-induced oxidized low-density lipoprotein uptake is class A scavenger receptor-dependent in macrophages. Prostaglandins Other Lipid Mediat..

[111] Westerterp M., Berbée J.F., Pires N.M., van Mierlo G.J., Kleemann R., Romijn J.A., Havekes L.M., Rensen P.C. (2007). Apolipoprotein C-I is crucially involved in lipopolysaccharide-induced atherosclerosis development in apolipoprotein E-knockout mice. Circulation.

[112] Engelmann M.G., Redl C.V., Nikol S. (2004). Recurrent perivascular inflammation induced by lipopolysaccharide (endotoxin) results in the formation of atheromatous lesions in vivo. Lab. Investig..

[113] An D., Hao F., Zhang F., Kong W., Chun J., Xu X., Cui M.Z. (2017). CD14 is a key mediator of both lysophosphatidic acid and lipopolysaccharide induction of foam cell formation. J. Biol. Chem..

[114] Boisvert W.A., Rose D.M., Johnson K.A., Fuentes M.E., Lira S.A., Curtiss L.K., Terkeltaub R.A. (2006). Up-regulated expression of the CXCR2 ligand KC/GRO-alpha in atherosclerotic lesions plays a central role in macrophage accumulation and lesion progression. Am. J. Pathol..

[115] Akhtar S., Hartmann P., Karshovska E., Rinderknecht F.A., Subramanian P., Gremse F., Grommes J., Jacobs M., Kiessling F., Weber C. (2015). Endothelial Hypoxia-Inducible Factor-1α Promotes Atherosclerosis and Monocyte Recruitment by Upregulating MicroRNA-19a. Hypertension.

[116] Gonçalves I., Edsfeldt A., Ko N.Y., Grufman H., Berg K., Björkbacka H., Nitulescu M., Persson A., Nilsson M., Prehn C. (2012). Evidence supporting a key role of Lp-PLA2-generated lysophosphatidylcholine in human atherosclerotic plaque inflammation. Arterioscler. Thromb. Vasc. Biol..

[117] Ketelhuth D.F., Bäck M. (2011). The role of matrix metalloproteinases in atherothrombosis. Curr. Atheroscler. Rep..

[118] Fan X., Wang E., Wang X., Cong X., Chen X. (2014). MicroRNA-21 is a unique signature associated with coronary plaque instability in humans by regulating matrix metalloproteinase-9 via reversion-inducing cysteine-rich protein with Kazal motifs. Exp. Mol. Pathol..

[119] Gu C., Wang F., Zhao Z., Wang H., Cong X., Chen X. (2017). Lysophosphatidic Acid Is Associated with Atherosclerotic Plaque Instability by Regulating NF-κB Dependent Matrix Metalloproteinase-9 Expression via LPA(2) in Macrophages. Front. Physiol..

[120] Bot M., de Jager S.C., MacAleese L., Lagraauw H.M., van Berkel T.J., Quax P.H., Kuiper J., Heeren R.M., Biessen E.A., Bot I. (2013). Lysophosphatidic acid triggers mast cell-driven atherosclerotic plaque destabilization by increasing vascular inflammation. J. Lipid Res..

[121] Mueller P.A., Yang L., Ubele M., Mao G., Brandon J., Vandra J., Nichols T.C., Escalante-Alcalde D., Morris A.J., Smyth S.S. (2019). Coronary Artery Disease Risk-Associated Plpp3 Gene and Its Product Lipid Phosphate Phosphatase 3 Regulate Experimental Atherosclerosis. Arterioscler. Thromb. Vasc. Biol..

[122] Aldi S., Matic L.P., Hamm G., van Keulen D., Tempel D., Holmstrøm K., Szwajda A., Nielsen B.S., Emilsson V., Ait-Belkacem R. (2018). Integrated Human Evaluation of the Lysophosphatidic Acid Pathway as a Novel Therapeutic Target in Atherosclerosis. Mol. Ther. Methods Clin. Dev..

[123] Oikonomou N., Mouratis M.A., Tzouvelekis A., Kaffe E., Valavanis C., Vilaras G., Karameris A., Prestwich G.D., Bouros D., Aidinis V. (2012). Pulmonary autotaxin expression contributes to the pathogenesis of pulmonary fibrosis. Am. J. Respir. Cell Mol. Biol..

[124] Vázquez-Medina J.P., Dodia C., Weng L., Mesaros C., Blair I.A., Feinstein S.I., Chatterjee S., Fisher A.B. (2016). The phospholipase A2 activity of peroxiredoxin 6 modulates NADPH oxidase 2 activation via lysophosphatidic acid receptor signaling in the pulmonary endothelium and alveolar macrophages. FASEB J..

[125] Chatterjee S., Feinstein S.I., Dodia C., Sorokina E., Lien Y.C., Nguyen S., Debolt K., Speicher D., Fisher A.B. (2011). Peroxiredoxin 6 phosphorylation and subsequent phospholipase A2 activity are required for agonist-mediated activation of NADPH oxidase in mouse pulmonary microvascular endothelium and alveolar macrophages. J. Biol. Chem..

[126] Sinclair K.A., Yerkovich S.T., Hopkins P.M., Fieuw A.M., Ford P., Powell J.E., O’Sullivan B., Chambers D.C. (2021). The autotaxin-lysophosphatidic acid pathway mediates mesenchymal cell recruitment and fibrotic contraction in lung transplant fibrosis. J. Heart Lung Transplant..

[127] Tager A.M., LaCamera P., Shea B.S., Campanella G.S., Selman M., Zhao Z., Polosukhin V., Wain J., Karimi-Shah B.A., Kim N.D. (2008). The lysophosphatidic acid receptor LPA1 links pulmonary fibrosis to lung injury by mediating fibroblast recruitment and vascular leak. Nat. Med..

[128] Funke M., Zhao Z., Xu Y., Chun J., Tager A.M. (2012). The lysophosphatidic acid receptor LPA1 promotes epithelial cell apoptosis after lung injury. Am. J. Respir. Cell Mol. Biol..

[129] Fernandez I.E., Eickelberg O. (2012). The impact of TGF-β on lung fibrosis: From targeting to biomarkers. Proc. Am. Thorac. Soc..

[130] Kramer E.L., Clancy J.P. (2018). TGFβ as a therapeutic target in cystic fibrosis. Expert. Opin. Ther. Targets.

[131] Huang L.S., Fu P., Patel P., Harijith A., Sun T., Zhao Y., Garcia J.G., Chun J., Natarajan V. (2013). Lysophosphatidic acid receptor-2 deficiency confers protection against bleomycin-induced lung injury and fibrosis in mice. Am. J. Respir. Cell Mol. Biol..

[132] Lucassen P.J., Fuchs E., Czéh B. (2004). Antidepressant treatment with tianeptine reduces apoptosis in the hippocampal dentate gyrus and temporal cortex. Biol. Psychiatry.

[133] Xu L., Su J., Guo L., Wang S., Deng X., Ma S. (2019). Modulation of LPA1 receptor-mediated neuronal apoptosis by Saikosaponin-d: A target involved in depression. Neuropharmacology.

[134] Lin M.E., Rivera R.R., Chun J. (2012). Targeted deletion of LPA5 identifies novel roles for lysophosphatidic acid signaling in development of neuropathic pain. J. Biol. Chem..

[135] Inoue M., Yamaguchi A., Kawakami M., Chun J., Ueda H. (2006). Loss of spinal substance P pain transmission under the condition of LPA1 receptor-mediated neuropathic pain. Mol. Pain..

[136] Velasco M., O’Sullivan C., Sheridan G.K. (2017). Lysophosphatidic acid receptors (LPARs): Potential targets for the treatment of neuropathic pain. Neuropharmacology.

[137] Ackerman S.J., Park G.Y., Christman J.W., Nyenhuis S., Berdyshev E., Natarajan V. (2016). Polyunsaturated lysophosphatidic acid as a potential asthma biomarker. Biomark. Med..

[138] Shi J., Jiang D., Yang S., Zhang X., Wang J., Liu Y., Sun Y., Lu Y., Yang K. (2020). LPAR1, Correlated With Immune Infiltrates, Is a Potential Prognostic Biomarker in Prostate Cancer. Front. Oncol..

[139] Decato B.E., Leeming D.J., Sand J.M.B., Fischer A., Du S., Palmer S.M., Karsdal M., Luo Y., Minnich A. (2022). LPA(1) antagonist BMS-986020 changes collagen dynamics and exerts antifibrotic effects in vitro and in patients with idiopathic pulmonary fibrosis. Respir. Res..

[140] Allanore Y., Distler O., Jagerschmidt A., Illiano S., Ledein L., Boitier E., Agueusop I., Denton C.P., Khanna D. (2018). Lysophosphatidic Acid Receptor 1 Antagonist SAR100842 for Patients With Diffuse Cutaneous Systemic Sclerosis: A Double-Blind, Randomized, Eight-Week Placebo-Controlled Study Followed by a Sixteen-Week Open-Label Extension Study. Arthritis Rheumatol..

[141] Zhao P., Yun Q., Li A., Li R., Yan Y., Wang Y., Sun H., Damirin A. (2022). LPA3 is a precise therapeutic target and potential biomarker for ovarian cancer. Med. Oncol..

[142] Tang X., Wuest M., Benesch M.G.K., Dufour J., Zhao Y., Curtis J.M., Monjardet A., Heckmann B., Murray D., Wuest F. (2020). Inhibition of Autotaxin with GLPG1690 Increases the Efficacy of Radiotherapy and Chemotherapy in a Mouse Model of Breast Cancer. Mol. Cancer Ther..

[143] Williams J.R., Khandoga A.L., Goyal P., Fells J.I., Perygin D.H., Siess W., Parrill A.L., Tigyi G., Fujiwara Y. (2009). Unique ligand selectivity of the GPR92/LPA5 lysophosphatidate receptor indicates role in human platelet activation. J. Biol. Chem..

[144] Wu W.T., Chen C.N., Lin C.I., Chen J.H., Lee H. (2005). Lysophospholipids enhance matrix metalloproteinase-2 expression in human endothelial cells. Endocrinology.

[145] Zhou Y., Little P.J., Ta H.T., Xu S., Kamato D. (2019). Lysophosphatidic acid and its receptors: Pharmacology and therapeutic potential in atherosclerosis and vascular disease. Pharmacol. Ther..

